# White matter alterations in MR-negative temporal and frontal lobe epilepsy using fixel-based analysis

**DOI:** 10.1038/s41598-022-27233-4

**Published:** 2023-01-02

**Authors:** Michaela Bartoňová, Jacques-Donald Tournier, Marek Bartoň, Pavel Říha, Lubomír Vojtíšek, Radek Mareček, Irena Doležalová, Ivan Rektor

**Affiliations:** 1 Central European Institute of Technology (CEITEC), Multimodal and Functional Neuroimaging Research Group, Masaryk University, Kamenice 753/5, 625 00 Brno, Czech Republic; 2 Brno Epilepsy Center, First Department of Neurology, St. Anne’s University Hospital, Faculty of Medicine, Masaryk University, Brno, Czech Republic; 3 Centre for Medical Engineering, King’s College London, London, UK; 4 Centre for the Developing Brain, King’s College London, London, UK

**Keywords:** Data processing, Statistical methods, Epilepsy, Epilepsy, Magnetic resonance imaging, Diffusion tensor imaging

## Abstract

This study focuses on white matter alterations in pharmacoresistant epilepsy patients with no visible lesions in the temporal and frontal lobes on clinical MRI (i.e. MR-negative) with lesions confirmed by resective surgery. The aim of the study was to extend the knowledge about group-specific neuropathology in MR-negative epilepsy. We used the fixel-based analysis (FBA) that overcomes the limitations of traditional diffusion tensor image analysis, mainly within-voxel averaging of multiple crossing fibres. Group-wise comparisons of fixel parameters between healthy controls (N = 100) and: (1) frontal lobe epilepsy (FLE) patients (N = 9); (2) temporal lobe epilepsy (TLE) patients (N = 13) were performed. A significant decrease of the cross-section area of the fixels in the superior longitudinal fasciculus was observed in the FLE. Results in TLE reflected widespread atrophy of limbic, thalamic, and cortico-striatal connections and tracts directly connected to the temporal lobe (such as the anterior commissure, inferior fronto-occipital fasciculus, uncinate fasciculus, splenium of corpus callosum, and cingulum bundle). Alterations were also observed in extratemporal connections (brainstem connection, commissural fibres, and parts of the superior longitudinal fasciculus). To our knowledge, this is the first study to use an advanced FBA method not only on the datasets of MR-negative TLE patients, but also MR-negative FLE patients, uncovering new common tract-specific alterations on the group level.

## Introduction

Since its first reported use in the 1850s, pharmacological treatment has remained the most efficient option for the majority of patients with epilepsy^1–3^. Although more than 20 different antiseizure drugs are currently in use and several others are the subject of intensive research^1^, only about 70% of pharmacologically treated patients with epilepsy achieve improved life quality and live seizure free^4^. When pharmacoresistance occurs in combination with no visible structural abnormalities on MRI (MR-negativity), this poses the greatest challenge for presurgical patient evaluation. Because the term ‘MR-negativity’ lacks a general definition, there is no clear line between MR-positive (visible lesion on MRI) and MR-negative epilepsy, and thus the same patient can be diagnosed as both MR-negative and MR-positive, depending on the sequences and field strength used. It has been shown that this line can be shifted by, for example, an upgrade from 1.5 to 3 T, and subsequently, to 7 T systems^5–7^. Ultra-high field MRI scanners, such as 7 T MRI, are usually not widely available for routine clinical practice. There is a rising need for other methods capable of detecting structural changes in MR-negative patients. Histopathological analyses of resected tissue (as resulting in seizure suppression) in patients diagnosed with MR-negative epilepsy^8^ have confirmed structural abnormalities resembling those found in patients with MR-positive epilepsy, such as focal cortical dysplasia (FCD), hippocampal sclerosis (HS), and gliosis. Since no study has yet confirmed a difference between MR-positive and MR-negative patients in terms of epilepsy duration, average number of seizures, age at disease onset, or other possible factors, the reason that some patients have visible abnormalities while others lack them remains unclear.

One modality that is beneficial in the study of epilepsy is diffusion-weighted imaging using MRI (dMRI). This modality has been widely used to describe alterations in white matter (WM) in patients diagnosed with epilepsy^9–11^. A large number of dMRI studies focused to some degree on homogenous sub-groups of MR-positive epilepsy patients, with temporal lobe epilepsy the most investigated^12,13^. However, it has not yet been completely addressed whether the same level and spatial characteristics of WM disruption might be in patients with MR-negative diagnoses as in MR-positive cases. Moreover, the majority of knowledge on WM impairment in epilepsy has been derived using diffusion tensor imaging (DTI), which comes with limitations^14^ that could be overcome by using advanced methods such as constrained spherical deconvolution (CSD)^15,16^ to model the diffusion in each voxel.

In this study, we present a retrospective group-specific analysis of the dMRI data of patients (as compared to a group of healthy controls) with focal pharmacoresistant epilepsy, from two patient groups: frontal (FLE) and temporal (TLE) lobe epilepsy. The epilepsy zone location was based on successful surgery after an MRI scan, with confirmation by histopathological examination and ILAE outcome Class ≤ 4 (at 12 months from surgery). We used an advanced method of CSD to model diffusion and the fixel-based analysis (FBA) method that produces one fibre measure per one fixel (= fibre element), with multiples placed in one voxel. This helps overcome limitations such as multiple fibres crossing in a voxel and the within-voxel averaging character of the conventional tensor model that has been used to derive the majority of current knowledge about the WM changes associated with epilepsy.

## Material and methods

### Participants

Based on a larger epilepsy cohort (see the overview of the complete initial cohort with the exclusion criteria described in the Supplementary Material, depicted in Fig. S2), 22 surgery candidates with MR-negative refractory epilepsy were included in the study: 13 patients later resected in the temporal lobe (3 left TLE, 10 right TLE) and 9 patients later resected in the frontal lobe (3 left FLE, 6 right FLE). In 3 T MRI scans conducted before surgery, MR-negativity was defined as no visible or inconclusive structural abnormalities. Protocol for MRI acquisitions to evaluate MR-negativity is listed in Supplementary Material Table S1. Patients subsequently underwent resective surgery, with target confirmed by the 12 months ILAE outcome Class ≤ 4. One hundred gender- and age-matched healthy individuals were recruited as a control group and written informed consent was obtained from all study participants. The study was approved by the Research Ethics Committee of Masaryk University and the Ethics Committee of St. Anne’s University Hospital and was designed in accordance with the Declaration of Helsinki. Detailed information about the subjects is provided in Table 1.

**Table 1:** Demographic and clinical summary of a patient group.

	TLE (N = 13)	FLE (N = 9)	HC (N = 100)
Gender	*7x* F, *6x* M	*7x* F, *2x* M	*48x* F, *52x* M
Age [± std]	34.86 y [± 9.48]	27.86 y [± 6.85]	31.65 y [± 8.35]
Avg no of seizures per month [± std]	5.67 y [± 4.97]	22.10 y [± 45.43]	–
Laterality	*10x* right *3x* left	*6x* right *3x* left	–
Disease duration [± std]	24.24 y [± 10.52]	14.52 y [± 6.70]	–
Onset age [± std]	10.62 y [± 9.50]	13.33 y [± 7.48]	–
Histology	*3x* negative^a^ *6x* HS *1x* FCD IIA *1x* FCD IIB *1x* FCD IIA + HS *1x* MA	*6x* FCD IIA *1x* FCD IIB *1x* FCD (unspecified) *1x* gliosis	–
ILAE (12 months)	*9x* ILAE 1 *1x* ILAE 3 *3x* ILAE 4	*3x* ILAE 1 *1x* ILAE 2 *2x* ILAE 3 *3x* ILAE 4	–

M, male; F, female; TLE, temporal lobe epilepsy; FLE, frontal lobe epilepsy; HC, healthy control; FCD, focal cortical dysplasia; HS, hippocampal sclerosis; y, years; MA, meningioangiomatosis.

^a^Patients with negative histological findings were included only if they achieved ILAE outcome ≤ 4.

Based on multimodal presurgical evaluation (positron emission tomography (PET), ictal and interictal single-photon emission computed tomography (SPECT–SISCOM), ictal and interictal video-EEG, high density scalp electroencephalography (EEG) and intracerebral stereoelectroencephalography (SEEG)) and neuropsychological testing, the Epilepsy Surgery Commission (including epileptologists, neurosurgeons, neuroradiologists, and psychologists with extensive experience) determined the surgery target.

### dMRI acquisition

Data were acquired prior to resection surgery on a 3 T Siemens MAGNETOM Prisma scanner. dMRI images were acquired using a 2 mm iso voxel, *b*-values = 700 s/mm^2^, 1000 s/mm^2^, 2300 s/mm^2^ with 30 diffusion directions per *b*-value shell. Furthermore, 10 repetitions for *b*-value = 0 s/mm^2^ were acquired using both phase-encoding directions (anterior–posterior and posterior-anterior).

### Diffusion image preprocessing

For preprocessing and analysis of dMRI data, Mrtrix3 3.0.3^17^, FSL 6.0.5^18^ (fsl.fmrib.ox.ac.uk) and ANTs 2.3.5^19^ were used. Preprocessing protocol consisted of noise correction using the Marchenko-Pastur principal component analysis (MP-PCA)^20,21^, Gibbs ringing artefacts correction^22^, susceptibility-induced distortion correction, correction for within-volume and between-volumes head motion, eddy currents correction and bias field correction^19^. The preprocessing quality was assessed by visual inspection and by FSL’s tool *eddyqc*^23^ with all subjects passing criteria.

### Fixel-based analysis and study-specific fibre orientation distribution template

From preprocessed data, the response functions were estimated for each tissue (WM, GM and CSF) separately using the Dhollander algorithm (*l*_max_ = 10)^24^. In order to increase anatomical contrast and enhance registration and statistical analysis, images were upsampled to 1.25 mm iso voxel. Then, one fibre orientation distribution function (FOD) was calculated per each voxel of WM using multi-shell multi-tissue constrained spherical deconvolution (*l*_max_ = 8; norm_lambda = 10^–10^; neg_lambda = 10^–10^) with the group-average response functions being kernels. The resulting FODs were corrected for bias fields and normalised for global intensity.

An unbiased symmetrical study-specific population template was created from FOD images and their left-to-right mirrored counterparts for a subset of 9 FLE, 9 TLE, and 9 HC, randomly selected subjects stratified for gender and age, and for epileptic side in patients. The mean squared error was used as a cost function. FOD images of all FLE (left/right: 3/6), TLE (left/right: 3/10), and HC subjects were subsequently registered to this template. For patients with left-lateralised epilepsy, flipped FOD images (right-to-left) were used with reoriented FOD lobes; the original FOD images were used for patients with right-lateralised epilepsy. The same proportion of flipped data was also used for the HC subjects (33% for subsequent FLE vs HC statistical analysis and 23% for subsequent TLE vs HC statistical analysis; subjects were chosen at random). Each subject’s FODs and the population template itself were visually assessed for spatial correspondence.

Three metrics were calculated per fixel (as described in^25^): apparent fibre density (FD)^26^, the fibre cross-section (FC, to ensure normal distribution of measure for a statistical comparison, logarithm of FC was calculated)^27^ and a combined measure of FD and FC (i.e. FDC) as their multiplication.

The 20 million streamlines whole-brain tractogram generated using probabilistic tractography of the template data was created and filtered to 2 million streamlines using the SIFT algorithm^28^. The template tractogram was used to generate the whole-brain fixel-fixel connectivity matrix.

### Fixel-based statistical analysis

A non-parametric one-tailed permutation t-test was used to compare whole brain FD, FC, and FDC between (1) 9 FLE vs 100 HC and (2) 13 TLE vs 100 HC. For both analyses, the groups were balanced for age, gender and mirror-wise flipping (more details in Supplementary Material Table S2) and the number of permutations was set to 5000. Age, gender, and whether the image had been flipped were used as covariates of no interest (design matrix condition numbers were 29.51 for FLE vs HC, and 25.55 for TLE vs HC). Only the decrease of all metrics in a group of patients, compared to HCs was tested. A threshold p = 0.05 with FWE correction using connectivity-based fixel enhancement^29^ was used.

The template tractogram has been segmented using TractSeg on 72 tracts (listed with used acronyms in Supplementary Material Table S3) and fixel masks of each tract has been produced and used to label the results of statistical analysis. By this approach, if e.g. voxel contained two fixels that corresponded to two different tracts crossing each other, one of those fixels being significantly altered, proper tract would be labelled.

### Ethical publication statement

We confirm that we have read the Journal’s position on issues involved in ethical publication and affirm that this report is consistent with those guidelines.

All subjects signed an informed consent form before entering the study. The study was approved by the Research Ethics Committee of Masaryk University and the Ethics Committee of St. Anne’s University Hospital. This study was designed in accordance with the Declaration of Helsinki.

## Results

### FLE vs HC

A significant reduction in FC was observed in FLE compared to HC, ipsilateral to the resected area in fixels of the dorsal and middle components of superior longitudinal fasciculus (SLF I, II).

### TLE vs HC

A significant reduction in FC and FDC was found in TLE compared to HC, ipsilateral to resected area in the superior cerebellar peduncle (SCP), corticospinal tract (CST), fronto-pontine tract (FPT), parieto-occipito-pontine tract (POPT), corticostriatal projection (striato-parietal, striato-postcentral, striato-precentral) and thalamocortical projection (thalamo-parietal, thalamo-postcentral, thalamo-precentral, thalamo-prefrontal, thalamo-premotor). Significant reduction was also seen in both parameters in the anterior and posterior midbody of the corpus callosum (CC4, CC5), splenium of corpus callosum (CC7), and bilaterally in superior thalamic radiation (STR).

Both FD and FDC parameters were reduced ipsilateral to the resection area within fixels corresponding to the cingulum bundle (CG), inferior fronto-occipital fasciculus (IFOF), uncinate fasciculus (UF), striato-fronto-orbital and striato-occipital projections. Additional reductions were found in both parameters in the fixels of the anterior commissure (AC) and contralaterally in the fixels of the superior cerebellar peduncle (SCP).

A reduction in FD exclusively was also found in the fixels of the ventral part of the SLF, ipsilaterally to resection side.

The majority of the significant results found contralateral to side of the resection were revealed using combined measure of FDC, in particular, reductions in the dorsal and ventral parts of the superior longitudinal fasciculus (SLF), corticospinal tract (CST), pontine tracts (fronto-pontine tract, parieto-occipito-pontine tract) and corticostriatal (striato-parietal, striato -postcentral, striato -precentral) and thalamocortical (thalamo-parietal, thalamo-postcentral, thalamo -precentral, thalamo -prefrontal, thalamo -premotor) projections. Additional reduction of FDC was found ipsilateral to the resection in anterior thalamic radiation (ATR) and optic radiation (OR), bilaterally in the fornix (FX) and in the fixels of the isthmus of corpus callosum (CC6).

**Figure 1:**
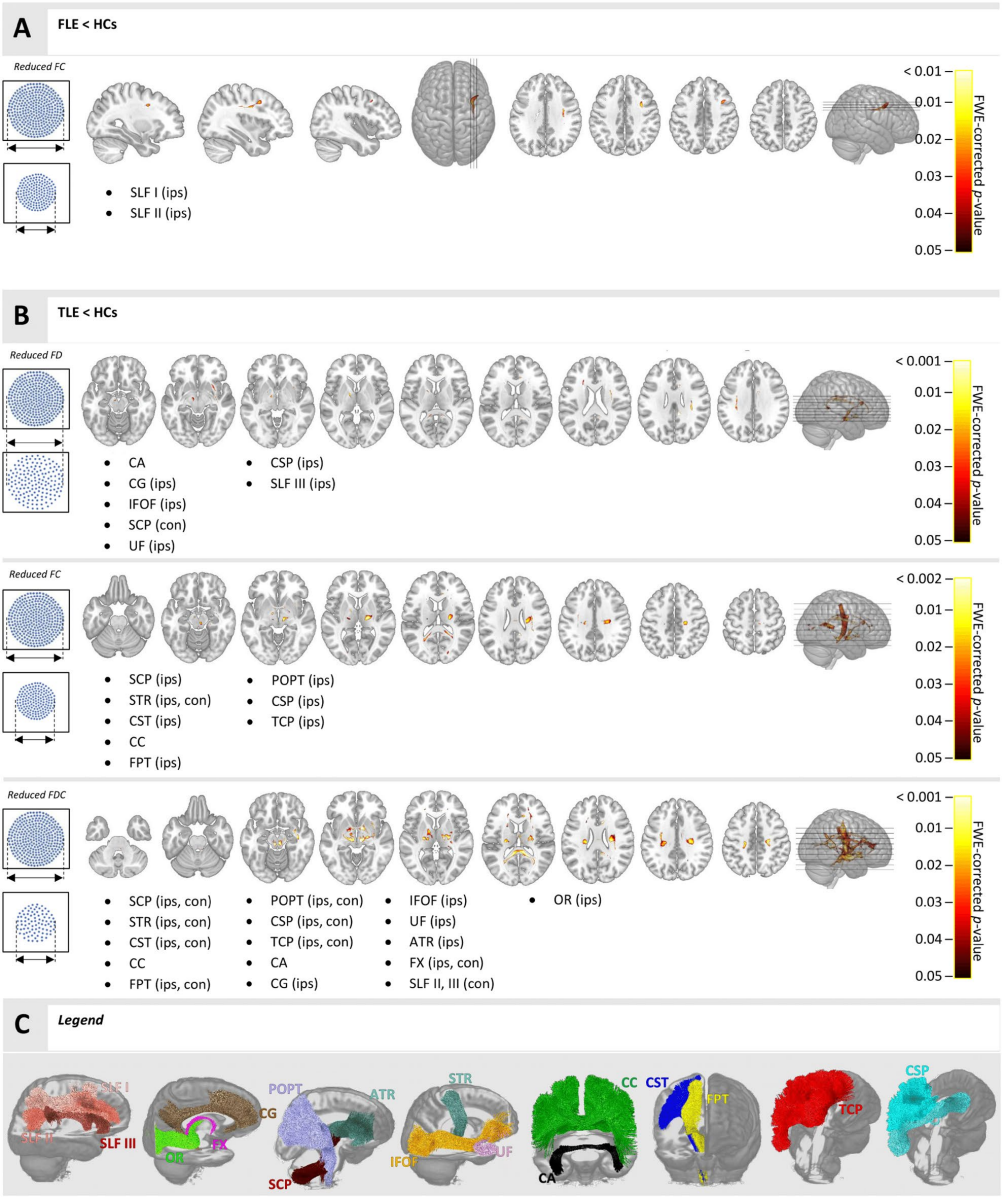
(**A**,**B**) Group analysis results—overlay of fixels with statistically significant effects (p < 0.05, FWE corrected) on the anatomical template (with listings of WM bundles corresponding to these fixels). (**A**) FLE vs HC statistical comparison in FC; (**B**) TLE vs HC statistical comparison in FD, FC, and FDC; (**C**) Complete 3D visualization of WM bundles with any previous significant results. FLE, frontal lobe epilepsy subjects; HC, healthy control subjects; TLE, temporal lobe epilepsy subjects; FD, fibre density; FC, fibre cross-section; FDC, fibre density and cross section; SLF, superior longitudinal fasciculus; CG, cingulum; OR, optic radiation; FX, fornix; STR, superior thalamic radiation; IFOF, inferior fronto-occipital fasciculus; UF, uncinate fasciculus; CST, corticospinal tract; FPT, fronto-pontine tract; POPT, parieto-occipito-pontine tract; ATR, anterior thalamic radiation; SCP, superior cerebellar peduncle; CA, anterior commissure; CC, corpus callosum (anterior midbody, posterior midbody, splenium and isthmus); TCP, thalamocortical projection; CSP, corticostriatal projection; ips, ipsilaterally to resection; con, contralaterally to resection Figure has been generated using mrview (MRtrix3 3.0.3^17^, https://www.mrtrix.org/) and tracts for Figure Legend were segmented using TractSeg^30^ (https://github.com/MIC-DKFZ/TractSeg).

Group analysis results are summarized in Table 2 and shown in Fig. 1 (more detailed, subject-specific plots of data from areas with significant between-group differences are in the Supplementary Material). Tracts with < 2% of significant fixels out of all fixels within the tract are not reported.

**Table 2:** Overview of a group statistical analyses that led to significant results.

	White matter tract	Percentage coverage			Type of the tract
		FD	FC	FDC	
FLE < HC	Superior longitudinal fasciculus I (ips)		2.14		Association
	Superior longitudinal fasciculus II (ips)		2.28		Association
TLE < HC	Superior cerebellar peduncle (ips)		10.31	16.72	Projection
	Superior thalamic radiation (ips)		24.91	26.35	Projection
	Superior thalamic radiation (con)		3.02	22.05	Projection
	Corticospinal tract (ips)		12.31	18.07	Projection
	Anterior midbody of corpus callosum		5.02	3.71	Commissural
	Posterior midbody of corpus callosum		7.20	5.54	Commissural
	Splenium of corpus callosum		4.54	9.77	Commissural
	Fronto-pontine tract (ips)		3.01	7.88	Projection
	Parieto-occipito-pontine tract (ips)		3.45	8.18	Projection
	Striato-parietal projection (ips)		2.23	6.25	Projection
	Striato-postcentral projection (ips)		4.12	8.39	Projection
	Striato-precentral projection (ips)		7.66	10.95	Projection
	Thalamo-parietal projection (ips)		2.79	5.29	Projection
	Thalamo-postcentral projection (ips)		9.76	11.82	Projection
	Thalamo-precentral projection (ips)		11.58	14.05	Projection
	Thalamo-prefrontal projection (ips)		2.03	3.42	Projection
	Thalamo-premotor projection (ips)		2.12	3.47	Projection
	Anterior commissure	4.13		14.82	Commissural
	Cingulum bundle (ips)	6.33		10.35	Association
	Inferior fronto-occipital fasciculus (ips)	2.20		5.86	Association
	Superior cerebellar peduncle (con)	2.24		18.99	Projection
	Uncinate fasciculus (ips)	2.51		6.93	Association
	Striato-fronto-orbital projection (ips)	2.02		3.80	Projection
	Striato-occipital projection (ips)	2.56		7.57	Projection
	Anterior thalamic radiation (ips)			4.35	Projection
	Fornix (ips)			38.43	Projection
	Fornix (con)			14.84	Projection
	Superior longitudinal fasciculus II (con)			2.84	Association
	Isthmus of corpus callosum			4.55	Commissural
	Optic radiation (ips)			2.55	Projection
	Superior longitudinal fasciculus III (con)			2.42	Association
	Corticospinal tract (con)			10.88	Projection
	Fronto-pontine tract (con)			8.59	Projection
	Parieto-occipito-pontine tract (con)			5.17	Projection
	Striato-parietal projection (con)			2.28	Projection
	Striato-postcentral projection (con)			5.50	Projection
	Striato-precentral projection (con)			5.73	Projection
	Thalamo-parietal projection (con)			4.34	Projection
	Thalamo-postcentral projection (con)			9.17	Projection
	Thalamo-precentral projection (con)			10.21	Projection
	Thalamo-prefrontal projection (con)			4.48	Projection
	Thalamo-premotor projection (con)			5.45	Projection
	Superior longitudinal fasciculus III (ips)	2.28			Association

HC, healthy control subjects; FD, fibre density; FC, fibre cross-section; FDC, fibre density and cross-section; ips, ipsilaterally to resection; con, contralaterally to resection.

## Discussion

In this study, diffusion MRI (dMRI) data of MR-negative patients with pharmacoresistant epilepsy who subsequently had resective surgery within the frontal or temporal lobes were studied. Choosing from a larger cohort of patients, we selected the two most prevalent subtypes of focal epilepsy^31–33^: TLE and FLE. WM impairment was investigated using fixel-based analysis (FBA), an advanced method for fibre property calculation. The aim of the study was to detect WM changes specific for those two types of MR-negative pharmacoresistant patients; WM changes were previously studied mainly on MR-positive homogenous subgroups of patients, predominantly diagnosed with TLE. Although there is not a sharp line between MR-positive and MR-negative epilepsy, it remains unclear why we are able to see structural changes in some patients and not in others. It is also important to note that our subjects are not a completely representative sample of MRI-negative focal epilepsy patients (for the selection process, see the Supplementary Material). The biggest limitation of this selection naturally comes from the specific design of the study—only data from patients with good surgery outcomes serve our purposes. This results in low sample sizes and underrepresents the whole pathology spectrum, as mainly FCD- and HS-related pathologies are represented. On the other hand, this approach allows us to work with a reliably identified epileptogenic focus, which is crucial for this type of analysis. This study aims to answer whether the disruption of WM pathways seen on MR-negative patients is analogous to the disruptions reported previously on MR-positive patients, using the advantage coming from an advanced dMRI analysis that goes beyond the commonly used diffusion tensor-based method. We calculated and compared three parameters for each fibre element (= fixel; with one voxel possibly containing multiples): fibre density (FD), fibre cross-section area (FC), and combined measure of fibre density and cross-section (FDC)^34^.

For patients with FLE (n = 9), a reduction in the fibre cross section was detected in the dorsal component of the superior longitudinal fasciculus (SLF I), the part connecting the superior and medial parietal cortices with the dorsal and medial parts of the frontal lobe, and in the middle component of the superior longitudinal fasciculus (SLF II), the bundle connecting the caudal-inferior parietal cortex and the dorsolateral prefrontal cortex^35^. The SLF I is involved in top-down attention and voluntary orientation of attention, as well as in working memory tasks; the SLF II is a WM bundle mainly important for motor control, but also attention, especially visuospatial awareness. Those changes are in accordance with previously reported fractional anisotropy (FA) reduction in patients with FLE-FCD^36^. To our knowledge, this is the first study to report FC reduction in the fixels of SLF I and SLF II in FLE patients with no structural abnormalities on MRI. Although the FLE group in our study was represented by patients with various seizure-onset locations within the frontal lobe, the common indicator for the group was FCD confirmed by histopathological examination in all nine patients. For this reason, the FLE patients were grouped together, despite different resection locations, as was done in previous studies of FLE^37^.

As expected from previously published studies^37,38^, much more widespread group-level differences were identified for TLE patients (n = 13) when compared to HC.

For TLE, changes in WM were detected predominantly, but not exclusively, ipsilateral to the seizure focus. The majority of WM fibre changes contralateral to resection were revealed using a combination measure of FD and FC; however, those changes were not significant using single parameters alone. This could suggest that changes within the WM of the hemisphere contralateral to resection are subtle when compared to those of the ipsilateral hemisphere. The majority of the fixels with decreased FBA metrics were located in the limbic connections (e.g. fornix), suggesting broader WM damage as a consequence of temporal epilepsy focus, and especially in the thalamic connections (anterior thalamic radiation, superior thalamic radiation, superior cerebellar peduncle, and other thalamo-cortical projections), with a higher prevalence on the side ipsilateral to seizure location. The role of the thalamus in seizure spread^39,40^ has been supported by successful electrical brain stimulation in the anterior thalamus in patients with epilepsy and by fMRI studies^41,42^. Concerning the corticostriatal connections, the inhibitory role of the basal ganglia (mainly the striatum and pallidum) in TLE seizure activity is already known^39,43,44^. The cortico-basal ganglia-thalamic circuit is believed to have an important role in regulating seizure propagation, whereas the impaired limbic circuit role is reflected in the neuropsychology of TLE. The typical manifestation of temporal seizure, also observed in our TLE subgroup, includes resurfacing of old memories or feelings of divergent emotions, together with sensory hallucination at the beginning of the seizure^45^—functions associated with the limbic system.

WM atrophy has also been revealed in other bundles directly connected to the temporal lobe (anterior commissure, inferior fronto-occipital fasciculus, uncinate fasciculus, splenium of corpus callosum, and cingulum bundle). These atrophy-related degenerations are probably driven by the propagation of seizures that originate in the temporal lobe, through brain networks to extratemporal regions.

Although brainstem connections are not a primary interest of epilepsy studies, we detected their altered microstructure (corticospinal tract, fronto-pontine tract, and parieto-occipito-pontine tract) mainly as a result of decreased fibre cross sections. Although rarely examined, brainstem connections have been receiving increased attention lately, mainly for their involvement in the ascending reticular activating system. Considering its role in consciousness, these connections could play a role in neurocognitive impairment in TLE epilepsy^46,47^.

Distinct alterations were observed in the extratemporal segments of the commissural fibres (anterior and posterior midbody of corpus callosum, isthmus of corpus callosum) and in the middle and ventral parts of the superior longitudinal fasciculus, a fibre interconnecting extratemporal areas. As a major interhemispheric pathway, the corpus callosum is believed to play a main role in seizure spread into the contralateral hemisphere. The involvement of extratemporal areas in pharmacoresistant TLE was reported in a meta-analysis of MRI-based volumetric studies^48^, and abnormal metabolic activity was detected using PET^49^ and in multiple fMRI studies^50^.

A possible explanation for the more extensive WM atrophy in TLE is the seizure character. Temporal lobe seizures are, in general, prolonged in comparison to the shorter (usually less than 30 s) frontal lobe seizures. The fact that the WM bundles of patients with TLE are exposed to the seizure propagation activity for a longer period of time could have a potentially degenerative impact on them, since it is well known that focal seizures drive fibre tract damage^25,38^. The TLE group in our study consisted of patients with either hippocampal sclerosis (HS) and/or focal cortical dysplasia (FCD) as revealed by postoperative histopathological examination. Although previous studies using DTI^31,37,51^ suggested that more extensive WM alterations are prevalent in TLE with HS, recent studies^31,52,53^ employing DTI, advanced FBA, and structural–functional connectivity have reported that FCD may be associated with changes of a similar extent, and many alterations did not differ between patients with and without HS. Assuming these facts, together with the low number of TLE patients naturally arising from the generally lower prevalence of our cohort (i.e. the prevalence of MRI-negative patients, relative to all epilepsy patients), these two histologically different groups were grouped together to avoid unreasonably small samples that would decrease statistical power if taken separately, so we focused on common WM alterations in these temporal lobe patients.

WM fibre bundle atrophy in TLE patients similar to that described here has been reported in multiple studies in DTI^37,54^ alteration (mainly a decrease of FA alongside other parameters such as radial and mean diffusivity) and FBA-derived^25,55,56^ parameters, on MR-positive patients with visible structural abnormalities. However, concerning the decline in FD, FC, and FDC that we were able to detect in areas distant from the temporal lobe, such as the contralateral corticospinal tract and superior longitudinal fasciculus, cerebellar connections through the superior cerebellar peduncle as well as atrophy in the corticostriatal and thalamo-cortical projections and in optic radiation are the first demonstration of WM alterations using FBA in a cohort of patients with very subtle to no changes on standard clinical MRI. Although non-lesional epilepsy patients are sometimes referred as lesional, only scanned using weaker magnetic fields (e.g. scanned on 3 T, whereas 7 T could reveal abnormalities), the name of the subgroup indicates that those patients lack grey matter (GM) alterations to the same extent as lesional patients, regardless of the disease duration or the seizure frequency. However, as demonstrated by our study, WM changes in this group are widespread and detectable using advanced dMRI methods, and the results are comparable (in terms of anatomical localization) to those of previous studies carried out on lesional patients. This suggests that this type of more sensitive analysis focused on WM may bring new insights in epilepsy patients with otherwise normal-appearing GM.

Although malformations in the resected areas were confirmed via histological examination, the biological basis of distant WM alterations remains unknown. Because of the acquisition settings, i.e. b values lower than 3000 s/mm^2^ (700 s/mm^2^, 1000 s/mm^2^, 2300 s/mm^2^) used in this study, intra-axonal, extra-axonal, and myelin compartments all potentially contributed to the estimated fibre densities. A potential explanation of the underlying histological alterations reflected in decreased FBA parameters in our study (and previously reported findings of reduced fractional anisotropy and increased mean and radial diffusivity parameters in other studies) could be myelin disruptions and overall declines in myelin content, increased extracellular space, or axonal degeneration in both myelinated and nonmyelinated types of bundles that were previously reported in patients with focal pharmacoresistant epilepsy^57–59^.

Fibre atrophy in the splenium of the corpus callosum, apparent from the reduction of all three observed parameters, is more probably the result of the drug treatment and seizures. DTI-based studies have shown that WM bundles of the splenium of the corpus callosum are more prone to structural abnormalities as a consequence of antiepileptic drug usage and they are also associated with seizure frequency, resulting in secondary WM degeneration^53,60–63^. Moreover, callosal fibres, cingulum bundles, inferior longitudinal fasciculus, arcuate fasciculus, and uncinate fasciculus have been reported to be associated with different seizure propagation directions (antero-posterior and medio-lateral spread), suggesting that a decrease in fibre microstructure parameters in these tracts is driven by seizure propagation rather than being a cause of seizures^54^. Whether the observed WM changes are a cause or a result, the amount of fibre structure abnormalities detected in the current and previous studies shows the importance of the role of WM in epilepsy—a disease that has for a long time been considered a disease of the GM only.

To conclude, our analysis of dMRI data from originally MR-negative TLE and FLE patients using a more precise fixel-level analysis instead of a voxel-level analysis revealed a widespread reduction of WM integrity parameters, extending beyond the lobes in which the seizures are generated. The majority of alterations seen in this study were previously reported on MR-positive epilepsy patients providing converging evidence for WM disruption in both MR-positive and MRI-negative epilepsy. To date, in-vivo non-invasive observations of WM microstructural changes in patients with epilepsy has been predominantly driven by the evidence from DTI-based studies of TLE with visible structural abnormalities during presurgical MRI. Although DTI is less demanding in terms of data acquisition (time, acquisition complexity/technical demands, etc.), we believe that more standard usage of CSD in cross-sectional clinical studies can enhance the knowledge on epilepsy previously gained mainly using oversimplified DTI. We encourage the acquisition of DWI data as High Angular Resolution Diffusion-Weighted Imaging (HARDI) data, even using 1.5 T clinical MRI, where time-consuming advanced acquisition can be compensated by e.g., Simultaneous Multi-Slice (SMS) acquisition. To the best of our knowledge, only a limited number of studies have utilized advanced diffusion analysis methods (as FBA or, e.g. fibre ball^64^), usually on MR-positive patients. This study benefits from employing more sensitive advanced FBA methods that enable the detection of even subtle pathological alterations of WM as compared to other conventional diffusion models. This is the first study to report tract-specific atrophy unique to patients with pharmacoresistant epilepsy that was performed on not only a group of MR-negative TLE patients, who represent the most common focal epilepsy syndrome^65^, but also on patients later successfully resected in the frontal lobe, experiencing focal seizures but having no visible or inconclusive structural preclinical abnormalities on 3 T MRI. Those changes were previously exclusively reported in patients with visible structural abnormalities.

## Supplementary Information

Supplementary Information.

## Data Availability

The data that support the findings of this study are available from the corresponding author upon reasonable request.
