# Supplementary material for: White matter alterations in MR-negative temporal and frontal lobe epilepsy using fixel-based analysis
Source: Sci Rep. 2023 Jan 2;13:19. doi: 10.1038/s41598-022-27233-4 (PMC9807578; doi:10.1038/s41598-022-27233-4)
Supplement: Supplementary file 1 — Supplementary Information. [file 41598_2022_27233_MOESM1_ESM.pdf]

White Matter Alterations in MR-Negative Temporal and Frontal Lobe Epilepsy Using Fixel-Based Analysis

Bartoňová Michaela, Tournier Jacques-Donald, Bartoň Marek, Říha Pavel, Vojtíšek Lubomír, Mareček Radek, Doležalová Irena, Rektor Ivan

Supplementary Material

Table S1: List of MRI sequences measured for evaluation of (non)lesionality of patients. TR = repetition time; TE = echo time; TI = inversion time; ms = milliseconds; mm = millimeters; deg = degrees.

**MRI sequence**	**No of slices**	**TR [ms]**	**TE [ms]**	**Voxel size [mm^3]**	**Flip angle [deg]**	**Notes**
**T2 – weighted** (turbo spin-echo)	Transversal	6100	105	0.49x0.49x3	150	
**T2* – weighted** (gradient-echo)	Transversal	852	19.9	0.69x0.69x3.9	20	transversal slices used to depict hemorrhage, calcification, and iron deposition
**T1 – weighted** (inversion recovery)	Transversal	2500	11	0.69x0.69x3.3	150	
**T2 – weighted** (turbo inversion recovery magnitude)	Transversal	5390	81	0.69x0.69x3.6	150	Long T1 components suppressed
**T1 – weighted** (inversion recovery)	Coronal	2900	10	0.5x0.5x2.2	150	
**T2 – weighted** (turbo inversion recovery magnitude)	Coronal	9000	81	0.69x0.69x3.6	150	
**T2 – weighted** (fast spin-echo)	Para-coronal	8000	52	0.39x0.39x2	150	Slices perpendicular to hippocampus
**T1 – weighted** (inversion recovery prepared 3D gradient-echo)	Sagittal	2300	2.33	1x1x1	8	TI = 900 ms
**T1 – weighted** (inversion recovery prepared 3D dual gradient-echo)	Sagittal	5000	2.97	1x1x1	(Dual flip angle) 1st: 4; 2nd: 5	TI_1 = 766 ms<br>TI_2 = 2500 ms
**T2 – weighted** (inversion recovery prepared 3D fast (turbo) spin-echo)	Sagittal	6000	387	1x1x1	–	TI = 1900 ms

Supplementary Methods

Description of the patient inclusion process:

The dataset for the current study was selected from a cohort recruited as part of a larger epilepsy project involving 154 epilepsy patients (see Figure S1) and 115 healthy control subjects (controls were excluded only if DWI data were missing or the acquisition was not finished, resulting in 100 subjects). From 154 patients, 40 were successfully resected (ILAE ≤4) and had at least a 12-month outcome from the surgery; the remaining 104 patients were either not yet resected, the surgery ended with ILAE outcome >4, or the follow up was shorter than 12 months. Out of 40 resected patients, 5 did not have DWI data or the acquisition was interrupted. From 35 patients, 30 were resected in the frontal or temporal lobe; the remaining 5 were resected in other areas (insular, parietal, or occipital). From 30 FLE/TLE patients, 3 were diagnosed as lesional (non-lesionality was first assessed according to 1.5T MRI data and then evaluated again using a 3T experimental MRI machine). The last exclusion resulted in 5 patients being omitted from the study: one patient was measured on a different MRI machine than the rest of our study group and the healthy controls group; one was excluded on the medical advice of his healthcare provider, as he did not follow the prescribed post-operative treatment regimen and therefore his outcome cannot be considered as a result of the operation; and three cases were excluded from the study for structural alterations incompatible with the methods used (two patients had substantial structural malformations not associated with epilepsy; one patient had a previous resection that resulted in structural changes).

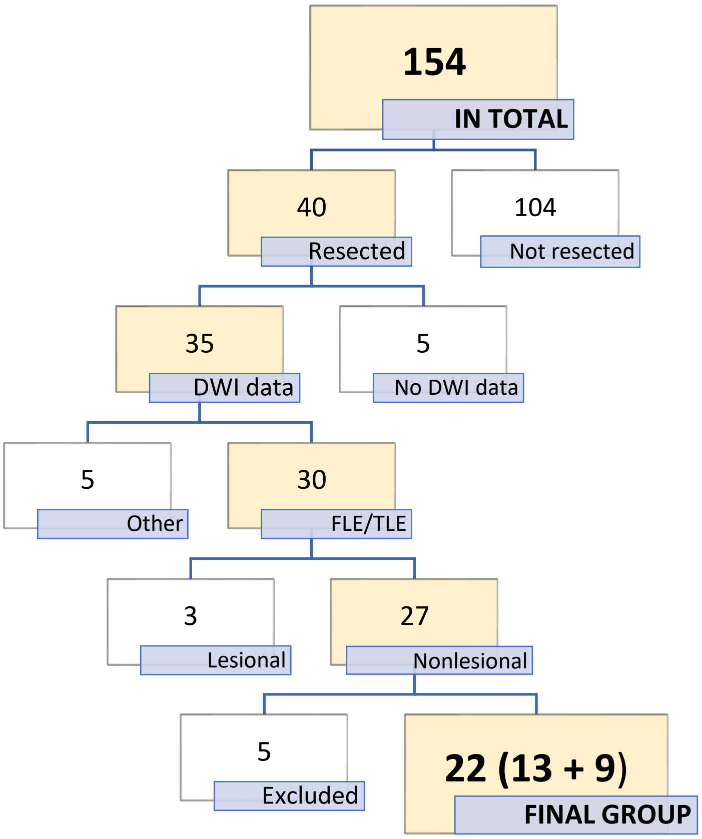

Figure S1: Overview of the whole initial cohort (marked as 'In total' in the figure) and the selection process resulting in the cohort used in the analyses (marked as 'Final group' in the figure). DWI = diffusion weighted imaging; FLE = frontal lobe epilepsy; TLE = temporal lobe epilepsy

Table S2: Overview of the demographic data of subject groups used for building a template and for statistical comparison. HC = healthy controls; SD = standard deviation; M = male; F = female; TLE = temporal lobe epilepsy; FLE = frontal lobe epilepsy

Template

	Gender [M/F]	Age [± SD]	Flip [flipped/original]
**HC (N=9)**	6/3	28.67 [±6.87]	9/9
**FLE (N=9)**	7/2	27.89 [±7.27]	9/9
**TLE (N=9)**	5/4	29.89 [±6.51]	9/9

For gender: $\chi^2 = 1$; $p = 0.6065$

For age: HC vs. FLE: 2-tailed *t*-stats = 0.2332; $p = 0.8186$; HC vs. TLE: 2-tailed *t*-stats = -0.3873; $p = 0.7036$; FLE vs. TLE: 2-tailed *t*-stats = -0.6149; $p = 0.5473$

For flip: $\chi^2 = 0$; $p = 1$

Statistical comparison FLE vs. HC

	Gender [M/F]	Age [± SD]	Flip [flipped/original]
**FLE (N=9)**	7/2	27.89 [± 7.27]	3/6
**HC (N=100)**	52/48	31.65 [± 8.39]	33/67

For gender: $\chi^2 = 2.2097$; $p = 0.1371$

For age: 2-tailed *t*-stats = -1.3001; $p = 0.1963$

Statistical comparison TLE vs. HC

	Gender [M/F]	Age [± SD]	Flip [flipped/original]
**TLE (N=14)**	6/7	34.92 [± 9.81]	3/10
**HC (N=100)**	52/48	31.65 [± 8.39]	23/77

For gender: $\chi^2 = 0.1574$; $p = 0.6916$

For age: 2-tailed *t*-stats = 1.2975; $p = 0.1971$

For flip: $\chi^2 = 0$; $p = 0.9951$

Table S3: List of WM tracts used for tractogram segmentation employing the TractSeg approach.

ID	ACRONYM	NAME OF A TRACT
**0**	AF_left	Arcuate fasciculus (right)
**1**	AF_right	Arcuate fasciculus (left)
**2**	ATR_left	Anterior thalamic radiation
**3**	ATR_right	Anterior thalamic radiation
**4**	CA	Anterior commissure
**5**	CC_1	Rostrum of corpus callosum
**6**	CC_2	Genu of corpus callosum
**7**	CC_3	Rostral body of corpus callosum
**8**	CC_4	Anterior midbody of corpus callosum
**9**	CC_5	Posterior midbody of corpus callosum
**10**	CC_6	Isthmus of corpus callosum
**11**	CC_7	Splenium of corpus callosum
**12**	CG_left	Cingulum bundle (left)
**13**	CG_right	Cingulum bundle (right)
**14**	CST_left	Corticospinal tract (left)
**15**	CST_right	Corticospinal tract (right)
**16**	MLF_left	Middle longitudinal fasciculus (left)
**17**	MLF_right	Middle longitudinal fasciculus (right)
**18**	FPT_left	Fronto-pontine tract (left)
**19**	FPT_right	Fronto-pontine tract (right)
**20**	FX_left	Fornix (left)
**21**	FX_right	Fornix (right)
**22**	ICP_left	Inferior cerebellar peduncle
**23**	ICP_right	Inferior cerebellar peduncle (right)
**24**	IFO_left	Inferior occipito-frontal fasciculus (left)
**25**	IFO_right	Inferior occipito-frontal fasciculus (right)
**26**	ILF_left	Inferior longitudinal fasciculus (left)
**27**	ILF_right	Inferior longitudinal fasciculus (right)
**28**	MCP	Middle cerebellar peduncle
**29**	OR_left	Optic radiation (left)
**30**	OR_right	Optic radiation (right)
**31**	POPT_left	Parieto-occipito-pontine tract (left)
**32**	POPT_right	Parieto-occipito-pontine tract(right)
**33**	SCP_left	Superior cerebellar peduncle (left)
**34**	SCP_right	Superior cerebellar peduncle (right)
**35**	SLF_I_left	Superior longitudinal fasciculus I
**36**	SLF_I_right	Superior longitudinal fasciculus I
**37**	SLF_II_left	Superior longitudinal fasciculus II
**38**	SLF_II_right	Superior longitudinal fasciculus II
**39**	SLF_III_left	Superior longitudinal fasciculus III
**40**	SLF_III_right	Superior longitudinal fasciculus III
**41**	STR_left	Superior thalamic radiation
**42**	STR_right	Superior thalamic radiation
**43**	UF_left	Uncinate fasciculus
**44**	UF_right	Uncinate fasciculus
**45**	CC	Corpus callosum
**46**	T_PREF_left	Thalamo-prefrontal projection (left)
**47**	T_PREF_right	Thalamo-prefrontal projection (right)
**48**	T_PREM_left	Thalamo-premotor projection (left)

**49**	T_PREM_right	Thalamo-premotor projection (right)
**50**	T_PREC_left	Thalamo-precentral projection (left)
**51**	T_PREC_right	Thalamo-precentral projection (right)
**52**	T_POSTC_left	Thalamo-postcentral projection (left)
**53**	T_POSTC_right	Thalamo-postcentral projection (right)
**54**	T_PAR_left	Thalamo-parietal projection (left)
**55**	T_PAR_right	Thalamo-parietal projection (right)
**56**	T_OCC_left	Thalamo-occipital projection (left)
**57**	T_OCC_right	Thalamo-occipital projection (right)
**58**	ST_FO_left	Striato-fronto-orbital projection(left)
**59**	ST_FO_right	Striato-fronto-orbital projection (right)
**60**	ST_PREF_left	Striato-prefrontal projection (left)
**61**	ST_PREF_right	Striato-prefrontal projection (right)
**62**	ST_PREM_left	Striato-premotor projection (left)
**63**	ST_PREM_right	Striato-premotor projection (right)
**64**	ST_PREC_left	Striato-precentral projection (left)
**65**	ST_PREC_right	Striato-precentral projection (right)
**66**	ST_POSTC_left	Striato-postcentral projection (left)
**67**	ST_POSTC_right	Striato-postcentral projection (right)
**68**	ST_PAR_left	Striato-parietal projection (left)
**69**	ST_PAR_right	Striato-parietal projection (right)
**70**	ST_OCC_left	Striato-occipital projection (left)
**71**	ST_OCC_right	Striato-occipital projection (right)

For a more detailed demonstration of the data in areas of significant between-group differences, see Figure S2 and the following overview of the results (with values representing group mean ± standard deviation):

A) FLE<HCs, parameter log(FC) – FLE: -0.0021±0.13; HC: 0.167±0.11
B) TLE<HC, parameter FD – TLE: 0.45±0.021; HC: 0.52±0.02
C) TLE<HCs, parameter log(FC) – TLE: -0.068±0.078; HC: 0.05±0.058
D) TLE<HCs, parameter FDC – TLE: 0.53±0.046; HC: 0.63±0.044

Data are adjusted for the same regressors as in whole-brain analyses, i.e. age, gender, and mirror-wise flipping.

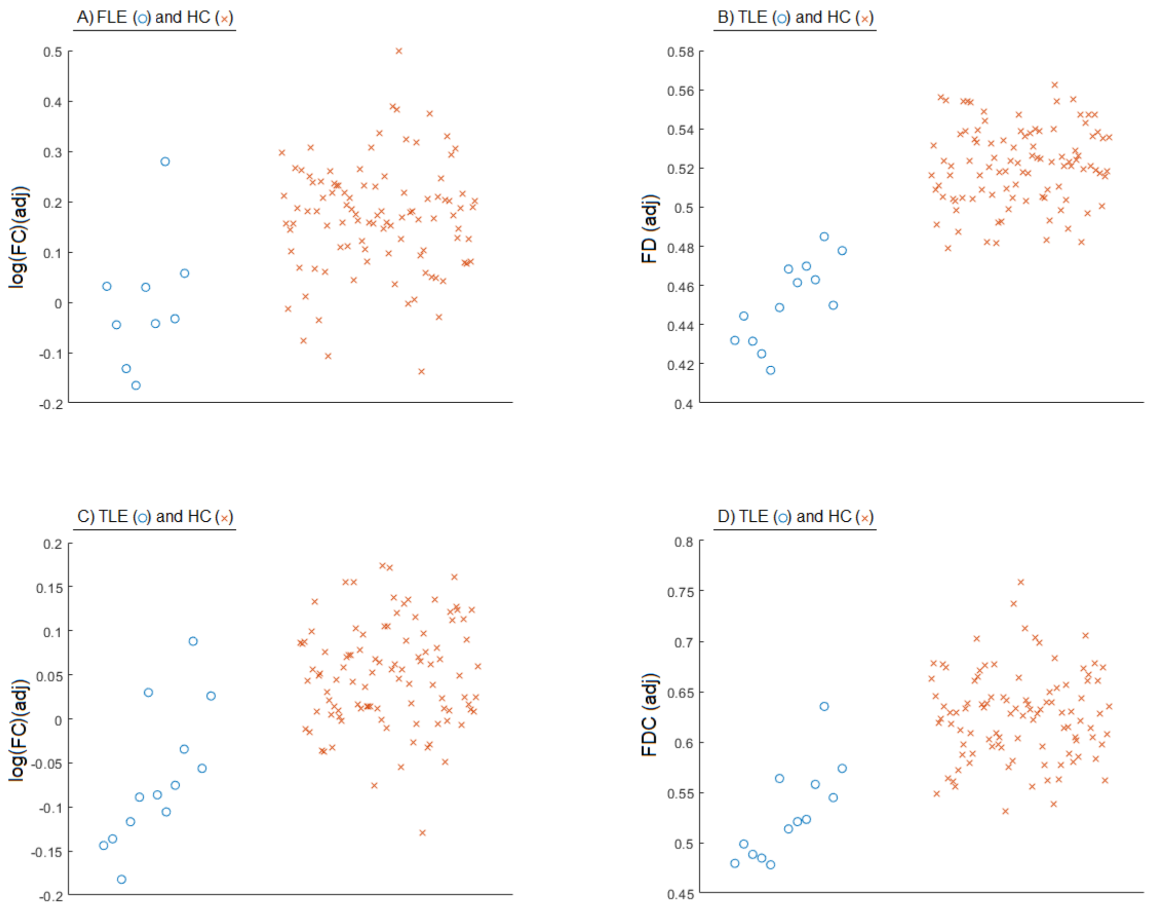

Figure S2: Overview of subject-specific data – mean value (one per subject) from areas with significant between-group differences in whole-brain analysis: A) FLE<HCs, parameter log(FC); B) TLE<HC, parameter FD; C) TLE<HCs, parameter log(FC); D) TLE<HCs, parameter FDC. HC = healthy controls; TLE = temporal lobe epilepsy; FLE = frontal lobe epilepsy; FD = fibre density; FC = fibre cross-section; FDC = fibre density and cross-section; adj =data adjusted for regressors (age, gender, and mirror-wise flipping).
